# Could AI risk perception, AI learning anxiety and personal growth initiative influence university students’ AI usage capability? An empirical investigation based on the control-value theory

**DOI:** 10.3389/fpsyg.2026.1788475

**Published:** 2026-03-18

**Authors:** Yali Jiang, Haiping Chen

**Affiliations:** Department of Psychology, Beijing Normal University, Beijing, China

**Keywords:** AI learning anxiety, AI risk perception, AI usage capability, personal growth initiative, the control-value theory

## Abstract

Based on the control-value theory, this study aims to explore the relationship among university students’ AI risk perception, AI learning anxiety, personal growth initiative, and AI usage capability. A questionnaire survey collected 1,001 valid data. SPSS 26.0 and PROCESS 3.4 were used for data processing. Results indicated that there was a significant negative correlation between AI risk perception and AI usage capability, and a significant indirect association through AI learning anxiety. Furthermore, personal growth initiative positively conditioned the negative association between AI risk perception and AI usage capability but negatively conditioned the negative relationship between AI learning anxiety and AI usage capability. This research offers a new perspective for understanding the emotional and cognitive mechanisms of college students in AI learning contexts and provides valuable insights for future AI education and system design.

## 1 Introduction

The rapid development of artificial intelligence (AI) has already begun to reshape the higher education ecosystem profoundly. AI has gradually become an important supporting tool for university students to complete course learning, information retrieval, writing, and research tasks. Both globally (Al Zaidy, 2024) and in China (Song et al., 2024), the prevalence of AI among university students has exceeded 60%. Moreover, many universities actively promote AI literacy education to help students improve their AI usage capability through courses, workshops, and learning support services (Xiao and Yi, 2021).

AI usage capability can not only enhance the efficiency of college students in completing learning tasks (Guan et al., 2025) but also further promote the development of their higher-order cognitive abilities such as critical thinking by reducing their learning burdens and optimizing their allocations of cognitive resources (Walter, 2024). However, existing studies treat AI usage capability as simply one dimension of AI literacy and neglect the independent value of AI usage capability itself as a key outcome. Research found that AI usage capability is the foundation of AI evaluation capability and even other higher-order abilities such as creativity6. In other words, without stable AI usage capability, it is difficult for learners to develop advanced evaluation and meta-cognition for AI. Therefore, it is necessary to separate AI usage capability from the AI literacy framework in order to investigate its key factors and mechanisms. Understanding the development of AI usage capability requires a theoretical framework that explains how learners’ AI perceptions give rise to emotional experiences and how these processes, shaped by individual differences in self-regulatory disposition, ultimately influence AI usage capability.

Previous research on the antecedents of AI usage capability has primarily focused on positive determinants such as perceived usefulness, ease of use, and trust, while paying limited attention to negative perceptual factors. AI risk perception can be conceptualized as a control-related appraisal cue that shapes individuals’ perceptions of manageability and uncertainty in AI-related learning contexts (Slovic et al., 2004; Goh et al., 2024). Such negative automatic appraisals of AI may not only affect individuals’ subsequent emotional experiences—such as AI learning anxiety—but also negatively effect the development of AI usage capability.

This study utilizes control–value theory (CVT) as the central framework to investigate the emotional mechanism of college students when learning and using AI tools. It examines how AI risk perception influences AI learning anxiety and subsequently impacts AI usage capability. Furthermore, it introduces personal growth initiative as a self-regulatory disposition and examines its conditional effects on both the direct and indirect association between AI risk perception and AI usage capability. By situating AI learning within the CVT framework, this study seeks to extend the theory’s application to technology-mediated learning contexts and to examine the regulatory mechanisms underlying AI-related emotions. It not only responds to Pekrun’s (2006, 2024) proposition that the theory can be used to explain various types of emotions but also provides new empirical support for the self-regulation mechanisms within the CVT framework. What’s more, this study enriches the theoretical connotation of personal growth initiative as a competence-oriented regulation strategy in new technology learning contexts and offers a new perspective for understanding individuals’ adaptation mechanisms under technological anxiety. Third, it also provides implications for AI literacy education in universities and AI system design.

The CVT was originally proposed to describe and explain emotions related to achievement in education8. In more recent work, the theory was extended into a broader framework with cross-domain applicability to epistemic, social, and existential emotions (Pekrun, 2006, 2024). According to this theory, emotions arise from individuals’ subjective appraisals of control (e.g., expectations for success) and value (e.g., perceived importance) in achievement-relevant activities. These emotions, in turn, influence cognitive resources, motivation, choice of learning strategy, and feedback processes, thereby shaping learning outcomes. Emotions include both those experienced during learning and those tied to outcomes. Positive achievement emotions generally promote adaptive engagement and better learning outcomes, whereas negative emotions tend to impede both. In addition, CVT emphasizes that individuals can engage in various forms of self-regulation, such as situation-oriented regulation and competence-oriented regulation, to manage the relationship between emotions and learning outcomes.

Some researchers have introduced CVT into AI-related research. Recent research investigated how teacher support reduces vocational college students’ AI learning anxiety by enhancing their self-efficacy (Chai and Sha, 2025). Others studies have treated AI as an environmental factor that can enhance perceived control and value through experimental methods, and examined the impact of AI usage on learners’ learning motivation, self-efficacy, anxiety, and interest (Chevalere et al., 2023; Shao, 2025). While these studies provide useful insights, they leave several theoretical gaps within the CVT framework. First, existing studies rarely conceptualize AI-related perceptions as appraisal antecedents; in particular, AI risk perception has not been explicitly positioned as a control-related appraisal cue within the CVT appraisal–emotion process. Second, prior CVT-based AI research has largely focused on emotions (e.g., anxiety, trust) as outcomes, leaving the appraisal–emotion–outcome pathway insufficiently tested for capability-related outcomes such as AI usage capability. Third, although CVT highlights the role of self-regulation—encompassing emotion-oriented, situation-oriented, appraisal-oriented, attention-oriented, and competence-oriented regulatory strategies—little is known about individual differences that predispose learners to engage in such regulatory efforts in AI learning contexts. Accordingly, this study aimed to investigate the association between AI risk perception, AI learning anxiety, personal growth initiative (PGI) and AI usage capability based on CVT. Specifically, drawing on CVT, the present study conceptualizes AI risk perception as a control-related appraisal cue, AI learning anxiety as an achievement emotion, AI usage capability as the focal learning outcome. Importantly, we conceptualize PGI as a self-regulatory disposition that shapes the extent to which, and the ways in which, individuals engage in specific regulatory strategies.

AI usage capability refers to an individual’s proficiency in leveraging the functions of AI tools to accomplish tasks efficiently (Wang et al., 2023). The core of this capability is operational proficiency, which enables users to utilize generative AI tools without in-depth thinking. This includes both an individual’s proficiency in using various functions of the same AI tool to complete complex tasks and the flexible application and integration of different AI tools to accomplish multiple tasks (An et al., 2025). According to CVT (Pekrun, 2006, 2024), the formation and development of the AI use ability are closely related to an individual’s perceived control and value of AI learning.

AI risk perception refers to an individual’s anticipation of potential adverse consequences from applying AI (Li, 2025), which is an overall evaluation of the sense of control and value regarding AI learning and use. CVT holds that during the process of generating and acquiring knowledge, individuals also experience emotions related to the cognitive characteristics of the knowledge (Pekrun, 2006, 2024). Confusion is one of these emotions. When existing information is contradictory or difficult for an individual to understand, confusion arises and becomes an important factor that leads individuals to underestimate the sense of control and value in learning activities (Lodge et al., 2018). In the context of learning and using AI, the “algorithmic black box” nature often heightens perceived risks, especially among university students who lack technical expertise. University students’ limited understanding of AI internal operating mechanisms and its potential ethical implications—such as academic integrity and originality—can amplify cognitive confusion and uncertainty (Rruplli et al., 2024). This confusion, in turn, diminishes learners’ trust and perceived control over AI tools, ultimately negatively influences their capability to effectively use AI in learning activities. Research also shows that AI risk perception can moderate the relationship between college students’ AI trust and AI acceptance. The higher the AI risk perception, the weaker the relationship between AI trust and AI acceptance (Zhang and Yu, 2025). Based on the above analysis, we may reasonably propose the hypothesis 1 that AI risk perception is negatively correlated with AI use ability.

According to CVT, there may be an associative pathway among AI risk perception, AI learning anxiety, and AI usage capability, in which AI learning anxiety is related to both variables. AI learning anxiety refers to the excessive fear caused by the changes brought about by artificial intelligence technology in personal or social life (Wu and Li, 2025). AI anxiety was classified into four dimensions: “Occupational replacement anxiety”, which refers to people’s concerns about the negative impacts of AI on workplace life; “Social - technological blind spots”, which refers to the anxiety arising from the failure to fully understand the dependence of AI on humans; “AI configuration anxiety”, which refers to the fear people have towards human—like AI; and “AI learning anxiety”, which refers to the anxiety people experience during the process of learning AI technology (Wang and Wang, 2022). This study aims to investigate the anxiety of college students during the process of learning and using AI, so only the dimension of AI learning anxiety is selected.

According to CVT, anxiety arises when individuals assign high negative value to potential failure outcomes while simultaneously perceiving low control over their success (Pekrun, 2006, 2024). In the context of AI learning, such anxiety may be positively correlated with individuals’ AI risk perception. Specifically, higher perceived AI risk may positively correlate with individuals’ evaluations of negative future outcomes, such as concerns about data privacy breaches, reduced personal creativity, or academic dishonesty—thus increasing the negative value appraisal of AI learning (Klein, 2025). In addition, higher perceived AI risk negatively associates with individuals’ control appraisals (Almaiah et al., 2022; Krieger et al., 2024), as learners might doubt their ability to use AI tools effectively or to ensure that AI-supported tasks could produce desirable outcomes. Consequently, both enhanced negative value and reduced perceived control, can jointly associate with increased AI learning anxiety, consistent with CVT (Pekrun, 2006, 2024).

Furthermore, CVT also holds that anxiety arising from an appraisal of high value but low control may, in rare cases, temporarily positively associates with extrinsic motivation, prompting individuals to invest more effort in the short term (Pekrun, 2006, 2024). However, a large body of theoretical and empirical research suggests that anxiety generally negatively correlates with learning processes and outcomes, even in AI areas (Chen et al., 2025; Pekrun, 2019). Anxiety consumes self-control resources and triggers task-irrelevant worries, thereby reducing the cognitive resources available for task processing and impairing information processing efficiency and problem-solving performance (Ward et al., 2020). What’s more, anxiety also tends to undermine intrinsic motivation and self-regulatory strategies, leading individuals to rely on rigid or overly analytical thinking patterns, which reduces cognitive flexibility and strategic shifting ability, thus hindering learning transfer and self-regulated learning (Du et al., 2022). Furthermore, studies have shown that task-irrelevant thoughts induced by anxiety not only prolong information processing time but also reduce knowledge acquisition during learning (Held et al., 2020; Theobald et al., 2022).

In the context of using AI, which are complex, require high cognitive load, and demand continuous learning (Ravšelj et al., 2025), AI anxiety may negatively correlate with individuals’ motivation to learn and use AI and resource allocation, which is not conducive to learners’ continuous use of AI (Li et al., 2025). Thus it may hinder the formation and development of their AI usage capability. Recent research found that AI anxiety significantly relates with negative attitudes towards AI use (Kaya and Çelebi, 2025). Another research showed that AI anxiety was related with individuals’ negative attitudes towards using AI for foreign language learning (Chen et al., 2024). According to the CVT, learners’ evaluation of control and value regarding learning activities directly influence their achievement emotions, which in turn affect learning outcomes (Pekrun, 2006, 2024). Accordingly, when learning to use AI, learners’ AI risk perception may affect their AI usage capability through AI learning anxiety. Therefore, we propose hypothesis 2 that there is an indirect association between AI risk perception and AI usage capability through AI learning anxiety.

According to CVT (Pekrun, 2006, 2024), individuals, as active agents, often proactively regulate their emotional perceptions during learning activities. Individuals enhance their personal abilities to increase the chances of success, thereby conditioning the relationship between emotions and learning outcomes (Pekrun, 2006, 2024). PGI may thus be an important conditioning variable for the indirect association between AI risk perception, AI learning anxiety, and AI usage capability. According to CVT (Pekrun, 2006, 2024), learners are active agents who do not merely experience achievement emotions but also regulate them through multiple strategies, including emotion-oriented, situation-oriented, appraisal-oriented, attention-oriented, and competence-oriented regulation. Within this framework, individual differences that predispose learners to engage in regulatory strategies should shape how appraisals and emotions translate into learning outcomes. Therefore, PGI (Green and Yıldırım, 2022) maybe an important conditioning variable for the indirect association between AI risk perception, AI learning anxiety, and AI usage capability.

PGI refers to an individual’s conscious and proactive tendency to improve himself (Robitschek, 1998). Individuals with high PGI often have a strong desire for self-improvement. They are skilled at identifying their development needs, planning actions, and actively putting those plans into practice (Jiao et al., 2024; Weigold et al., 2020). Research on university students has shown that the higher an individual’s personal growth initiative, the higher his level of occupational engagement (Gong et al., 2022). In AI learning contexts, when individuals perceive higher AI-related risks, those high in PGI are more likely to engage in adaptive regulatory strategies. For example, they may employ appraisal-oriented regulation by reframing AI-related risks as manageable challenges, situation-oriented regulation by proactively seeking guidance, resources, or supportive learning environments, and competence-oriented regulation by investing effort in building AI-related knowledge and skills. These regulatory efforts should help maintain or enhance capability development despite elevated risk perceptions. Thus, PGI is expected to buffer the adverse association between AI risk perception and AI usage capability. We therefore propose hypothesis 3 that personal growth initiative positively conditions the association between AI risk perception and AI usage capability such that the negative correlation between AI risk perception and AI usage capability is stronger when personal growth initiative is low.

PGI may also conditions the later path between AI learning anxiety and AI usage capability. Previous studies have shown that PGI is an important moderator in the relationship between stress and mental health in adolescents, with PGI buffering the negative impact of stress on an individual’s mental health (Zaman and Naqvi, 2018). Research during COVID-19 has also indicated that PGI can moderate the relationship between fear and life satisfaction (Green and Yıldırım, 2022). However, there is currently insufficient evidence regarding whether personal growth initiative can moderate the relationship between anxiety and learning outcomes. According to CVT (Pekrun, 2006, 2024), PGI may condition the association between AI learning anxiety and AI usage capability. Individuals high in PGI may be more likely to enact adaptive regulatory strategies when experiencing AI learning anxiety. From a CVT perspective, they may engage in attention-oriented regulation by redirecting attention from threat-related cues to task-relevant information, and in emotion-oriented regulation by managing anxiety rather than being overwhelmed by it. Such regulatory efforts help conserve cognitive resources and reduce self-control depletion associated with anxiety (Weigold et al., 2020), thereby allowing individuals to remain focused on AI learning activities (Held and Heubeck, 2025). In addition, individuals high in PGI are inclined toward competence-oriented and problem-solving regulation, actively investing effort in skill development and seeking solutions to learning challenges. Through proactive behaviors such as setting realistic goals, gathering relevant information, and persistently implementing growth plans, PGI can enhance perceived self-efficacy and control (Cai and Lian, 2022; Weigold et al., 2020). Strengthened self-efficacy further supports sustained engagement and capability development despite experiencing anxiety. Taken together, by facilitating multiple forms of regulation and protecting motivational and cognitive resources, PGI is expected to buffer the detrimental impact of AI learning anxiety on AI usage capability. Therefore, we propose Hypothesis 4 that personal growth initiative positively conditions the association between AI learning anxiety and AI usage capability such that the negative correlation between AI learning anxiety and AI usage capability is stronger when personal growth initiative is low.

**Figure 1:**
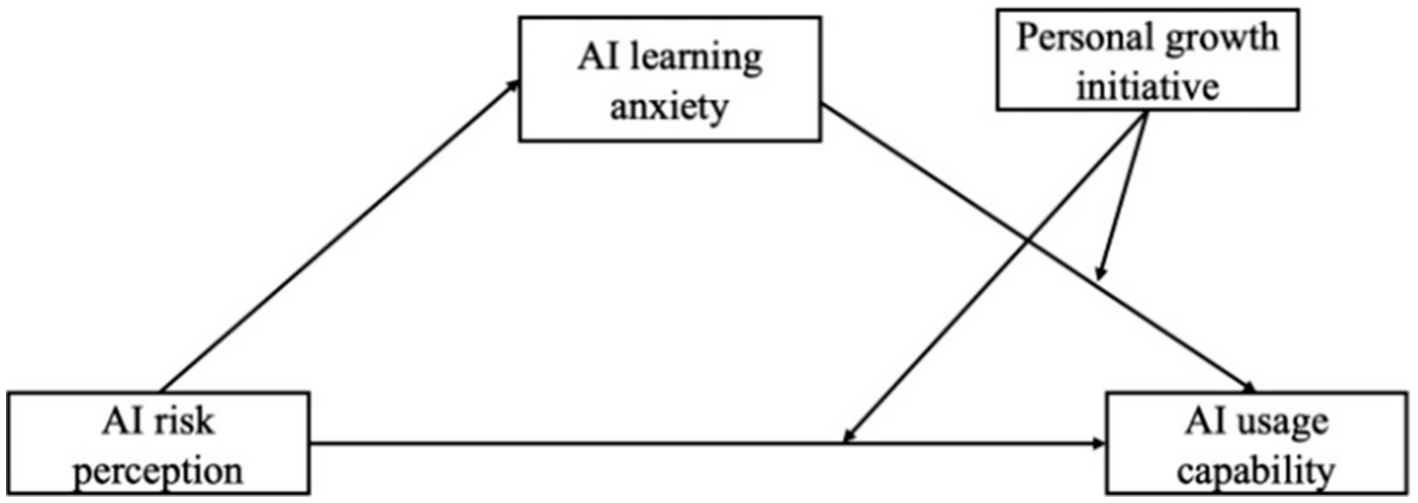
The proposed model.

In summary, the present framework conceptualizes the relationships among AI risk perception, AI learning anxiety, AI usage capability, and PGI as a conditional process model. Specifically, AI risk perception is expected to influence AI usage capability both directly and indirectly through AI learning anxiety, while personal growth initiative conditions the strength of both the direct path and the anxiety–capability link. Consequently, the indirect effect of AI risk perception on AI usage capability is expected to vary as a function of personal growth initiative. This structure aligns with a moderated mediation model (Hayes, 2018), which enables a more precise test of the CVT-based mechanism whereby appraisals shape achievement emotions and, in turn, learning outcomes, with self-regulatory dispositions influencing the strength of these associations. Accordingly, PROCESS Model 15 was employed to examine the conditional indirect effects proposed in this study (see Figure 1).

## 2 Methods

### 2.1 Ethical statement and participants

A convenience sampling method was used on undergraduates from a university in Jiangsu, China in November, 2025. A questionnaire link was generated using Credamo, a globally well-known data collection platform. The college administrators send the questionnaire link to students and ask them to fill it out during breaks or other leisure time. Each questionnaire was filled out anonymously to protect the privacy of the participants. The participants could withdraw from the survey at any time, and they were informed that the questionnaire was not related to their academic performance. Finally, 1,001 valid questionnaires were obtained. Females accounted for 76.4%, and the average age of the participants was 19.26 (1.48). In terms of academic disciplines, 32.9% of the participants were from humanities-related majors, 22.7% from social sciences, 8.5% from science and engineering disciplines, and 36.0% from other fields. In addition, the study assessed participants’ usage frequency across six types of AI tools, including Generative AI (e.g., ChatGPT), literature search tools (e.g., ResearchRabbit), literature reading tools (e.g., Zotero), data-related tools (e.g., GitHub Copilot), AI-assisted writing tools (e.g., Paperpal), and other AI tools. The results indicated that Generative AI were the most frequently used tools, with 82.0% of participants reporting regular use. This was followed by literature reading tools (25.7%) and literature search tools (24.1%).

Ethical approval for this study was obtained from the Institutional Review Board of Beijing Normal University (IRB No. BNU202601050002). All participants gave their informed consent electronically before taking part in the study.

### 2.2 Measures

The research team translated the original English items into Chinese. The translated items were carefully reviewed to maintain conceptual equivalence. During data collection, participants did not report ambiguity or comprehension difficulties. The PGIS-II was administered using an established Chinese version.

The AI learning anxiety subscale was used, which contained 8 items (Wang and Wang, 2022). The items were scored with a seven-point Likert-type rating scale (1 = never through 7 = completely) for questions such as “Learning to use specific functions of an AI technique/product makes me anxious.” The Cronbach’s *α* was 0.92.

Three questions were used to measure AI risk perception, namely security, privacy, and output errors (Zhang and Yu, 2025). The items were also scored with a seven-point Likert-type rating scale (1 = Strongly Disagree 7 = Strongly Agree) for questions such as “In the process of using AI, one’s personal information or data may be collected and misused.” Here the Cronbach’s α was 0.90.

The Personal Growth Initiative Scale–II (PGIS-II) was employed to assess personal growth initiative (Robitschek et al., 2012). Items are rated on a 6-point Likert scale ranging from 1 (strongly disagree) to 6 (strongly agree). For example: “When I try to change myself, I actively seek help.”. The Cronbach’s α of the scale was 0.90.

Finally, three items were used to measure AI usage capability (Wang et al., 2023). These items were scored with a 5-point Likert scale ranging from “Strongly Agree” to “Strongly Disagree” For questions such as “I can skillfully use AI applications to help me with my daily learning.” The Cronbach’ α of this scale was 0.72.

### 2.3 Statistical analysis

All statistical analyses were conducted using SPSS 27.0. Exploratory factor analysis (EFA) and confirmatory factor analysis (CFA) were conducted to examine the factor structure of the scales. Both analyses were performed using the same dataset (*N* = 1,001). Although it is methodologically preferable to conduct EFA and CFA on independent samples or randomly split subsamples for cross-validation purposes, the relatively large sample size in the present study ensured stable parameter estimation. First, a common method bias test was performed to examine potential bias associated with self-report measures. Descriptive statistics and correlation analyses were then conducted for all study variables. No missing data were observed, as the online survey platform required mandatory responses for all items.

Prior to hypothesis testing, main variables were standardized to reduce potential multicollinearity in interaction analyses. Multicollinearity diagnostics indicated that variance inflation factor (VIF) values ranged from 1.42 to 1.75, well below the recommended threshold of 5, suggesting no multicollinearity concerns. Examination of residual plots indicated no serious violations of linearity or homoscedasticity assumptions.

Hypotheses were tested using the PROCESS macro (Models 4 and 15). A bootstrap procedure with 5,000 resamples was employed to estimate 95% confidence intervals (CIs). Effects were considered significant when the 95% CI did not include zero. The use of bootstrapping enhances the robustness of mediation analysis by avoiding normality assumptions of the indirect effect.

### 2.4 Results

#### 2.4.1 Measurement model validation

Given the context-sensitive nature of the constructs, an exploratory factor analysis (EFA) was initially served to support the validity of the translated items, as EFA is commonly used in Chinese contexts to verify whether the items align with the intended theoretical constructs. The KMO value was 0.96, and Bartlett’s test of sphericity value was 32555.72 (*p* < 0.001). Furthermore, the structure explained 76.79% of the total variance in the model. Table 1 indicates the factor loadings of the items. Combined, these results suggest that this solution was a reasonable interpretation of the present survey (see Table 1).

**Table 1:** Exploratory factor analysis results (only factor loadings greater than 0.5 are shown).

Variables	Component			
	1	2	3	4
AI risk perception1	0.70			
AI risk perception 2	0.64			
AI risk perception 3	0.67			
AI usage capability1		0.58		
AI usage capability2		0.54		
AI usage capability3		0.51		
AI learning anxiety1			0.78	
AI learning anxiety2			0.83	
AI learning anxiety3			0.85	
AI learning anxiety4			0.83	
AI learning anxiety5			0.85	
AI learning anxiety6			0.84	
AI learning anxiety7			0.85	
AI learning anxiety8			0.79	
PGI1				0.77
PGI2				0.82
PGI3				0.82
PGI4				0.84
PGI5				0.83
PGI6				0.79
PGI7				0.83
PGI8				0.83
PG9				0.83
PGI10				0.85
PGI11				0.84
PGI12				0.85
PGI13				0.85
PGI14				0.85
PGI15				0.85
PGI16				0.84

Subsequently, a confirmatory factor analysis (CFA) was conducted to rigorously test the measurement model obtained from the EFA (Anderson and Gerbing, 1988). The measurement model was assessed by goodness-of-fit, construct reliability and construct validity. Goodness-of-fit was determined based on GFI, CFI, TLI, RMSEA. All model fit statistics were within the acceptable ranges (*χ*^2^/df = 3.24 < 5, GFI = 0.92 > 0.90, CFI = 0.97 > 0.90, TLI = 0.95 > 0.90, RMSEA = 0.065 < 0.08, SRMR = 0.063 < 0.1) (Hair et al., 2010), which demonstrates that the measurement model exhibits satisfactory values.

Construct reliability and validity were assessed using multiple indicators. Composite reliability (CR) was used to evaluate internal consistency, while convergent validity was examined through the average variance extracted (AVE). All CR values were greater than 0.70, demonstrating acceptable reliability. The AVE values for all constructs exceeded the recommended threshold of 0.50, indicating satisfactory convergent validity (Fornell and Larcker, 1981). As presented in Table 2, the square roots of AVE were compared with the inter-construct correlations to assess discriminant validity. All correlations were lower than the corresponding AVE square roots, supporting adequate discriminant validity. Overall, these results indicate that the proposed measurement model demonstrates satisfactory reliability and validity (see Table 3).

**Table 2:** Results of discriminant validity.

Variables	AI usage capability	AI risk perception	AI usage capability	Personal growth initiative
AI learning anxiety	0.89			
AI risk perception	0.31	0.85		
AI usage capability	−0.31	−0.39	0.71	
Personal growth initiative	−0.19	−0.53	0.61	0.85

**Table 3:** Results of construct reliability and validity.

Variables	Convergent validity AVE	Composite reliability CR
AI learning anxiety	0.80	0.97
AI risk perception	0.72	0.89
AI usage capability	0.51	0.73
Personal growth initiative	0.72	0.97

#### 2.4.2 Common method bias test

To address potential common method bias, this study employed both procedural and statistical remedies. During data collection, procedural controls such as anonymous responses were implemented to reduce respondents’ evaluation apprehension and response tendencies. Statistically, the unmeasured latent method factor approach proposed by Podsakoff et al. (2003) was applied. The results indicated that adding the common method factor did not substantially improve model fit (ΔCFI = 0.001, ΔTLI = 0.001, ΔRMSEA = 0.001). Therefore, common method bias is unlikely to pose a serious threat to the validity of the study’s conclusions.

#### 2.4.3 Descriptive statistics and correlation analysis

As can be seen from Table 4, there is a significant positive correlation between AI risk perception and AI learning anxiety (*r* = 0.31, *p* < 0.01), a significant negative correlation between AI risk perception and AI usage capability (*r* = −0.40, *p* < 0.01), and a significant negative correlation between AI learning anxiety and AI usage capability (*r* = −0.31, *p* < 0.01).

**Table 4:** Descriptive statistics and correlations between variables.

Variables	*M*	SD	1	2	3
AI risk perception	3.80	1.19	1		
AI learning anxiety	4.72	1.27	0.31**	1	
AI usage capability	3.25	0.66	−0.40**	−0.31**	1
Personal growth initiative	3.88	0.89	−0.53**	−0.20**	0.61**

*p < 0.05; **p < 0.01.

#### 2.4.4 Indirect association analysis

The analysis was conducted using Model 4 in PROCESS v3.4. The results are presented in Table 5.

**Table 5:** Analysis results of the indirect role of AI learning anxiety.

Variables	AI usage capability		AI learning anxiety		AI usage capability	
	*β*	*t*	*β*	*t*	*β*	*t*
Constants	3.25**	17.66	0.000	0.00	3.25**	174.70
AI risk perception	−0.26**	−13.64	0.31**	10.25	−0.22**	−11.12
AI learning anxiety					−0.14**	−6.99
*R* ^2^	0.16		0.09		0.20	
*F*	*F* (1,999) = 186.21**		*F* (1,999) = 105.14**		*F* (2,998) = 122.04**	

*p < 0.05; **p < 0.01.

AI risk perception had a significant total effect on AI usage capability [*β* = − 0.26, *p* < 0.01, 95%CI (−0.29, −0.22)], supporting hypothesis 1. Moreover, AI risk perception significantly and positively correlated with AI learning anxiety (*β* = 0.31, *p* < 0.01). When both AI risk perception and AI learning anxiety were entered into the regression equation, AI learning anxiety showed a significant negative effect with AI usage capability (*β* = − 0.14, *p* < 0.01), while the direct effect of AI risk perception on AI usage capability remained significant (*β* = − 0.22, *p* < 0.01). Bootstrap analysis with 5,000 resamples indicated that the indirect effect of AI risk perception on AI usage capability through AI learning anxiety was −0.04, with a 95% confidence interval of [−0.10, −0.04], excluding zero. This result confirms a significant partial indirect effect, suggesting that AI learning anxiety partially mediates the relationship between AI risk perception and AI usage capability, supporting hypothesis 2.

#### 2.4.5 Conditioning test

**Figure 2:**
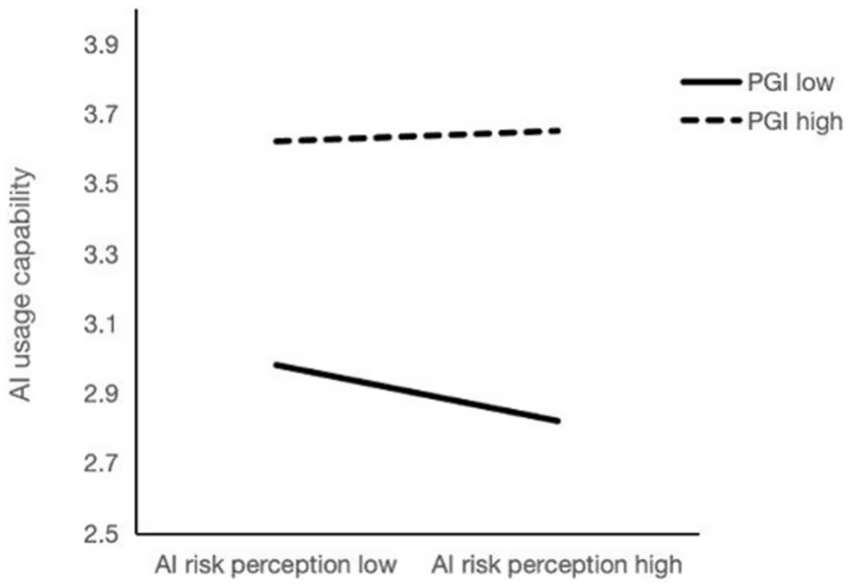
The conditioning effect of PGI on the relationship between AI risk perception and AI usage capability. PGI, personal growth initiative.

The analysis was conducted using Model 15 in PROCESS v3.4. The results are presented in Table 6. The index of conditioning indirect effect was −0.02, with a standard error of 0.01 and a 95% confidence interval of [−0.03, −0.01], indicating that PGI significantly conditioned the indirect effect of AI learning anxiety on AI usage capability. The interaction between AI risk perception and PGI significantly correlated with AI usage capability [*β* = 0.31, *p* < 0.01, 95% CI (0.02, 0.07)]. This indicates that PGI positively conditioned the relationship between AI risk perception and AI usage capability. Conditional direct effect analysis further revealed that when PGI was low (−1 SD), AI risk perception significantly and negatively predicted AI usage capability [*β* = − 0.08, p < 0.01, 95% CI (−0.12, −0.03)]. However, when PGI was high (+1 SD), the effect of AI risk perception on AI usage capability was non-significant [*β* = 0.02, *p* > 0.05, 95% CI (−0.03, 0.06)]. These findings suggest that higher levels of PGI may negatively with the negative association between AI risk perception and AI usage capability (see Figure 2).

**Table 6:** Analysis results of the conditioning role of PGI.

Variables	AI usage capability		AI learning anxiety	
	*β*	*t*	*β*	*t*
Constants	1.66**	19.82	0.000	0.00
AI risk perception	−0.24**	−4.06	0.31**	10.25
AI risk perception*PGI	0.415**	19.69		
AI learning anxiety	0.05**	3.81		
AI learning anxiety*PGI	−0.05**	−3.58		
*R* ^2^	0.43		0.09	
*F*	*F* (5,995) = 149.41**		*F* (1,999) = 105.14**	

PGI, personal growth initiative, *p < 0.05; **p < 0.01.

**Figure 3:**
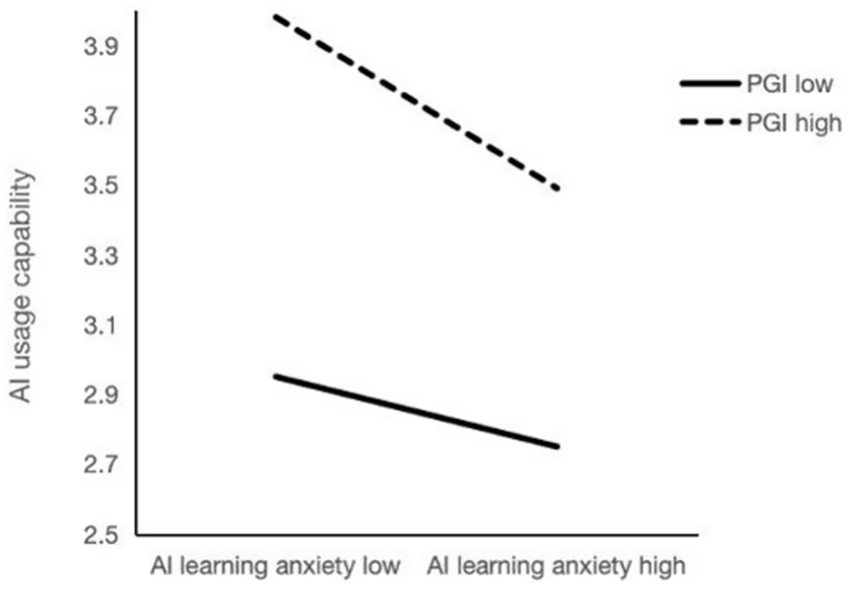
The conditioning effect of PGI on the relationship between AI learning anxiety and AI usage capability. PGI, personal growth initiative.

The interaction between AI learning anxiety and PGI significantly correlated with AI usage capability [*β* = − 0.05, *p* < 0.01, 95% CI (−0.07, −0.02)], indicating that PGI negatively conditioned the relationship between AI learning anxiety and AI usage capability, not supporting hypothesis 4. Conditional indirect effect analysis further showed that when PGI was low (−1 SD), the effect of AI learning anxiety on AI usage capability was non-significant [*β* = −0.02, *p* > 0.05, 95% CI (−0.04, 0.01)]. However, when personal growth initiative was high (+1 SD), AI learning anxiety significantly and negatively predicted AI usage capability [*β* = −0.05, *p* < 0.01, 95% CI (−0.07, −0.03)]. These results suggest that higher PGI positively associated with the negative association between AI learning anxiety and AI usage capability (see Figure 3).

## 3 General discussion

### 3.1 Discussion

This study investigated the relationship between AI risk perception, AI learning anxiety, and AI usage capability using a questionnaire survey of 1,001 college students. The results show that there was a significant negative correlation between AI risk perception and the ability to use AI. That is, when an individual perceives a higher level of AI risk, his own capability to use AI is lower. This result is consistent with previous research and also conforms to the basic assumptions of control-value theory. It found that the threat assessment of AI can affect an individual’s willingness to adopt it (Kinskofer and Tulis, 2025). Individuals’ perceptions of AI risks negatively correlates with their willingness to adopt the technology (Kenesei et al., 2022). However, the dependent variables examined in these studies are basically attitudes and acceptance of AI usage rather than AI usage capability (Wei et al., 2025). Compared to previous studies, the contribution of this research lies in linking an individual’s perception of AI risks with his own capability to use it.

The results revealed a significant partial indirect effect of AI learning anxiety on the relationship between AI risk perception and AI usage capability, which suggests that AI risk perception affects AI usage capability not only indirectly through anxiety but also through additional pathways that are not fully captured by emotional processes. According to CVT (Pekrun, 2006, 2024), emotions are generated from individuals’ cognitive appraisals of control and value, which subsequently influence learning motivation and outcomes. In this context, AI learning anxiety serves as one important emotional factor linking perceived AI risk to performance outcomes a result that is also consistent with previous research findings (Zhang et al., 2022). The partial mediation effect also suggests the possible existence of other mediating variables. According to CVT (Pekrun, 2006, 2024), anxiety is a future-oriented, high-arousal, negative outcome emotion that exerts a complex influence on learning outcomes. Therefore, other variables such as hope may also serve as potential mediators in this relationship.

PGI significantly conditioned the direct association between AI risk perception and AI usage capability, such that the negative relationship was weaker among individuals with higher PGI. Within the CVT framework, AI risk perception can be conceptually interpreted as primarily reflecting diminished control appraisal—namely, perceptions of limited capability or manageability in dealing with AI-related learning and usage demands. When individuals perceive AI as risky, they may experience reduced control over task requirements and anticipated outcomes. In addition, heightened risk perception may increase perceived costs and uncertainty. Because cost constitutes an integral component of subjective task value within CVT, elevated perceived costs may correspond with diminished value appraisal (Pekrun, 2024). Accordingly, learning activities appraised as low in control and value are less likely to foster effective engagement and performance (Pekrun, 2024). From this perspective, higher AI risk perception may negatively affect AI usage capability by signaling diminished control and reduced task value in technology-related activities. PGI may condition this relationship by strengthening individuals’ regulatory engagement and competence-oriented strategy. Individuals high in PGI are typically characterized by proactive goal-setting, deliberate self-regulation, and strong developmental commitment (Xiao et al., 2025). Such characteristics may help sustain perceived control when confronting technological uncertainty and complexity. Although control and value appraisals were not directly measured in the present study, this explanation should be understood as a theoretically grounded interpretation rather than a directly tested mechanism. Prior research linking PGI to enhanced self-efficacy and adaptive engagement in challenging contexts provides indirect support for this appraisal-based account (Junça-Silva and Lourenço, 2025; Liao et al., 2024).

Interestingly, we found a negative moderating effect of PGI on the relationship between AI learning anxiety and AI use capability. Specifically, when personal growth initiative was higher, the detrimental impact of AI learning anxiety on AI use capability became stronger. Consistent with the preceding discussion, individuals high in PGI are distinguished by an achievement-oriented mindset, active self-regulation, and a disciplined orientation toward developmental tasks (Xiao et al., 2025). They tend to maintain elevated self-regulatory standards and strong control-oriented expectations regarding their ability to manage developmental challenges effectively. According to CVT, achievement emotions are shaped by perceived control and value appraisals (Pekrun, 2024). Individuals high in PGI typically hold strong control-oriented expectations and elevated self-regulatory standards (Jiao et al., 2024). In contrast, AI learning anxiety reflects perceived insufficient control over AI-related demands and, as a future-oriented and highly activated negative emotion (Kraft et al., 2023; Saviola et al., 2020), signals uncertainty about one’s capability to cope with complex technological challenges while requiring substantial regulatory resources. For individuals high in PGI, the experience of anxiety may therefore create a discrepancy between their strong control expectations and their perceived loss of control. This expectancy–emotion mismatch can intensify self-focused attention and increase efforts to restore control, thereby heightening cognitive load. When regulatory demands exceed available resources, greater self-control depletion may occur, ultimately amplifying the detrimental effect of anxiety on AI usage capability. In this sense, the negative moderating effect of PGI aligns with CVT’s emphasis on control appraisals: stronger control-oriented standards may paradoxically magnify the disruptive impact of anxiety when perceived control is low.

Therefore, although PGI is generally regarded as an adaptive motivational resource, its beneficial effects may depend on the alignment between perceived control and emotional experience. In technology-intensive learning environments characterized by ambiguity and rapid change, strong control-oriented standards may paradoxically exacerbate the disruptive influence of anxiety.

### 3.2 Practical implications

The results of this study have significant implications for the designers of artificial intelligence systems and school educators. Designers of AI systems should pay special attention to how to explain the risk of AI to users in order to minimize the AI learning anxiety of nonprofessional users (Balalle and Pannilage, 2025). First, they should set up a risk statement or model behavior explanation module in the human-computer interaction interface to guide users to deeply understand the algorithm mechanism of AI and reduce users’ concerns about the “algorithm black box.” Second, on the start interface, various functions of AI should be displayed in a visual way such as operation videos to reduce users’ anxiety about learning and using AI. Third, clear instructions should be established for the regular updates of AI products. Designers should inform users of the connections and differences between the latest features, and help them reduce their learning burdens. Fourth, considering the sensitivity of women to AI learning anxiety, targeted prompt and explanations can be set to help female users evaluate AI more reasonably.

Additionally, school educators, such as library educators, should pay attention to the following points when developing, setting up and configuring a series of courses and resources to enhance students’ AI usage capability. First, educators should incorporate contents such as the limitations of AI, and black box nature into the curriculum to enable students to have more realistic expectations and avoid unnecessary anxiety. Second, educators should pay special attention to incorporating academic norms and restrictions on AI usage to prevent students from relying solely on AI and thus avoiding academic misconduct issues. This can also reduce students’ risk and anxiety about using AI (Zhang and Xu, 2025). Third, attention should be paid to students’ confidence in using AI. Scaffolding should be set up, and students’ confidence in using AI should be enhanced through activities such as templates, examples, step-by-step exercises, and peer support. Fourth, educators can design balanced intervention strategies that combine competence-oriented training (e.g., self-efficacy enhancement, problem-solving skills) with emotion-oriented guidance (e.g., coping with uncertainty). This dual approach may prevent over activation of control motivation and promote healthier engagement with AI technologies.

### 3.3 Limitations and future research directions

Although this study has achieved some meaningful results, the following limitations still exist. First, although this study proposed a mediation pathway, the causal direction should be interpreted with caution due to the cross-sectional nature of the data. The hypothesized sequence was theoretically grounded in the assumption that perceived risk may trigger affective responses (e.g., anxiety), which in turn could hinder the development of usage capability. However, alternative explanations are equally plausible. Prior studies suggest that competence-related factors—such as AI literacy or usage capability—may themselves influence affective reactions such as anxiety. Therefore, the present model reflects a theoretical assumption rather than a confirmed causal process. Future research should employ longitudinal or experimental designs to clarify the temporal ordering and reciprocal dynamics among these constructs.

Second, this study only investigated college students and did not include postgraduate students, researchers, or working groups, which reduces the generalizability of the research results. Future research can expand the diversity of samples by selecting samples from across regions, schools, and even industries (Granić, 2024). In addition, although the participants were drawn from a comprehensive university encompassing multiple faculties and academic disciplines, all respondents were recruited from the same institution. This single-institution sampling strategy may introduce contextual bias related to institutional culture, thereby constraining the broader applicability of the results. Future studies are encouraged to employ multi-institutional or cross-regional sampling designs to enhance external validity and reduce potential selection bias.

Third, this study examined only AI learning anxiety, a future-oriented, high-arousal negative emotion, and found it to be only a partial mediator. Future research should further explore other emotions that differ in temporal orientation, object focus, activation level, and valence. Fourth, the negative moderating effect of personal growth initiative observed in this study suggests the presence of other contextual factors. Future research should therefore further investigate variables that moderate the effect of PGI and compare emotion-oriented and competence-oriented regulation strategies to examine their similarities and differences.

Fourth, the sample consisted predominantly of female participants (76.4%), which may limit the generalizability of the findings. Prior research suggests that gender differences may exist in technology-related anxiety and usage behaviors (Cai et al., 2017). Therefore, the current results should be interpreted with caution. Future studies are encouraged to recruit more gender-balanced samples to examine whether the observed relationships are robust across different gender groups.

Finally, EFA and CFA were conducted on the same sample rather than on independent samples or randomly split subsamples. Although the large sample size enhances statistical stability, this approach may limit the strictly confirmatory nature of the CFA and increase the possibility of overfitting. Future research should cross-validate the factor structure using independent samples to further strengthen construct validity.

## 4 Conclusion

This study explored the impact of AI risk perceptions and AI learning anxiety on college students’ AI usage capability, with a focus on the moderating role of PGI. Our results reveal a “double-edged sword” effect of PGI: while PGI buffers the negative impact of AI risk perception on AI usage capability, it simultaneously amplifies the negative relationship between AI learning anxiety and AI usage capability. This dual role of PGI offers new insights into the emotional and cognitive mechanisms involved in AI learning, particularly in the context of competence-oriented regulation as outlined in CVT. Specifically, our findings refine the understanding of how PGI interacts with emotional responses like anxiety, thereby influencing students’ ability to use AI effectively. This study contributes to the theoretical framework of CVT by highlighting the context-dependent, dual role of PGI in moderating anxiety and risk perception. Additionally, it addresses the call for more research on individual traits in understanding AI usage. The study also offers nuanced implications for AI education practice, suggesting that fostering PGI can have both positive and negative consequences depending on the learner’s emotional state. Future AI educational programs should aim to balance the benefits of promoting PGI with strategies for managing anxiety, ensuring that students are equipped to engage with AI technologies effectively.

## Data Availability

The original contributions presented in the study are included in the article/supplementary material, further inquiries can be directed to the corresponding author.
